# ID 41 - Qualidade de Pareceres de Resposta Rápida Produzidos por Profissionais de Secretarias Estaduais de Saúde após Programa de Treinamento em Avaliação de Tecnologias em Saúde

**DOI:** 10.5327/2237-9622.2025.v34s1.41

**Published:** 2025-11-25

**Authors:** Marina Petrasi Guahnon, Gilson Pires Dorneles, Ana Paula Blankenheim, Bruna Marmett, Rodrigo Pereira de Almeida, Bárbara Cristiane da Silva, Suena Medeiros Parahiba, Maicon Falavigna

**Keywords:** Avaliação de Tecnologias em Saúde, processo de tomada de decisão, sistema de saúde pública

## Abstract

**Introdução:**

A Avaliação de Tecnologias em Saúde (ATS) é um campo emergente nas Secretarias Estaduais de Saúde (SES), e que requer treinamento de recursos humanos para apoiar os processos de tomada de decisão. Portanto, há uma necessidade de estratégias educacionais para aprimorar as habilidades profissionais e disseminar práticas baseadas em evidências científicas. Dessa forma, o objetivo é relatar e descrever a qualidade do trabalho final desenvolvido pelos profissionais de 27 SES do País participantes do Programa de Treinamento em ATS.

**Método:**

Servidores de 27 SES participaram de um programa de treinamento em ATS, que consistiu na realização de uma trilha de cursos on-line, com carga horária total de 140 horas e distribuídos entre as áreas de "Introdução à ATS", "Saúde Baseada em Evidências" e "Avaliação Econômica em Saúde", além de um oficina presencial, com carga horária total de 25 horas, para realização de uma atividade prática sobre o tema. Ao final do programa, cada SES foi orientada a desenvolver um parecer de resposta rápida focado em intervenções para alguma condição de saúde de interesse. A qualidade desses documentos foi submetida a uma avaliação por meio da ferramenta do Instituto Joanna Briggs. A avaliação foi realizada por um pesquisador externo não envolvido com o curso e os dados foram apresentados de forma qualitativa.

**Resultados:**

Das 27 SES participantes, todas concluíram todo o Programa de Treinamento e 24 (88,9%) entregaram o documento com a atividade final. Destaca-se que esta foi a primeira experiência em uma atividade em ATS realizada por vários dos representantes das SES. Dos 24 documentos produzidos, 21 (87,5%) apresentaram uma questão de revisão de forma clara e transparente, atendendo ao item 1 da lista de verificação ("A questão da revisão foi claramente e explicitamente formulada?"). Um total de 17 documentos (70,83%) atenderam ao item 10 da lista de verificação ("Os dados apresentados apoiam as práticas recomendadas e as políticas?") ao relatar informações que sustentam recomendações de práticas e políticas, 13 dos 24 pareceres elaborados (54,16%) atenderam aos itens 2 ("Os critérios de elegibilidade para a inclusão dos estudos foram apropriados?") e 4 ("As fontes para a busca de estudos foram adequadamente utilizadas?") da lista de verificação ao usar adequadamente os critérios de elegibilidade e as fontes para busca de estudos na revisão, respectivamente. O item que obteve a menor pontuação da lista de verificação foi o item 9 ("O viés de publicação dos estudos incluídos foi avaliado e tratado?"), pois apenas 1 trabalho acessou o viés de publicação dos estudos incluídos (4,17%).

**Conclusão:**

Os dados descritos anteriormente demonstram que o formato utilizado pelo programa pode ser uma estratégia eficaz para o treinamento profissional nessa área, proporcionando recursos educacionais para pesquisadores em ATS no âmbito estadual em todas as regiões do Brasil. No entanto, são necessárias mais ações de treinamento e aperfeiçoamento em ATS para o contexto estadual de saúde pública.

